# Low-dose histrelin for ovulation induction in mares: a partially randomized crossover pilot study

**DOI:** 10.1007/s11259-026-11583-9

**Published:** 2026-10-20

**Authors:** Lys de Barros Fogagnoli, Kallena Canali Abdala Jose, Rachel Weckl, Clara Mantovani Ozorio Mota, Mirelle Ochrimowicz Fernandes, Rafaela Caroline Machado, Carlos Eduardo Camargo, Pedro Vicente Michelotto

**Affiliations:** 1 Graduate Program in Animal Science, School of Medicine and Life Sciences, Pontifícia Universidade Católica do Paraná, Rua Imaculada Conceição 1155, Prado Velho, Curitiba, Paraná 80215-901 Brazil; 2 Veterinary Program, School of Medicine and Life Sciences, Pontifícia Universidade Católica do Paraná, Rua Imaculada Conceição 1155, Prado Velho, Curitiba, Paraná 80215-901 Brazil

**Keywords:** Acupuncture, Equine, Horse, Reproduction, Welfare

## Abstract

This partially randomized crossover pilot study evaluated reduced doses of histrelin for ovulation induction in mares and compared intramuscular (IM) administration with pharmacopuncture at the *Bai-hui* and *Yan-chi* acupoints. Eleven mares were enrolled, of which nine completed all eight protocols and were included in the analyses. The protocols comprised 250 µg histrelin IM (standard dose), 75 µg IM, 25 µg IM, no intervention, saline at *Bai-hui*, saline at *Yan-chi*, 25 µg histrelin at *Bai-hui*, and 25 µg histrelin at *Yan-chi*. Ovulation was assessed by transrectal ultrasonography at scheduled examinations until detected, with ovulation within 48 h as the primary outcome. Ovulations first detected at 24 h were excluded from the primary analysis. Ovulation within 48 h occurred in 88.9% (8/9) of observations with 250 µg IM, 87.5% (7/8) with 75 µg IM, 87.5% (7/8) with 25 µg IM, 37.5% (3/8) with no intervention, 50.0% (3/6) with saline at *Bai-hui*, 100% (9/9) with 25 µg histrelin at *Bai-hui*, 12.5% (1/8) with saline at *Yan-chi*, and 87.5% (7/8) with 25 µg histrelin at *Yan-chi*. Exact 95% confidence intervals were wide, reflecting the small sample size. Exploratory pairwise comparisons with the no-intervention protocol were performed without adjustment for multiple comparisons and should be interpreted cautiously. The findings provide preliminary evidence that reduced histrelin doses, including 25 µg, may induce ovulation within 48 h in mares. However, this study did not demonstrate an advantage of pharmacopuncture over IM administration of the same reduced dose. Larger, fully randomized studies are required to confirm the efficacy of reduced-dose histrelin and determine whether administration at specific acupoints provides any additional effect.

**Supplementary Information:**

The online version contains supplementary material available at https://doi.org/10.1007/s11259-026-11583-9.

## Introduction

Histrelin is a synthetic gonadotropin-releasing hormone (GnRH) agonist that stimulates the release of luteinizing hormone (LH) and follicle-stimulating hormone (FSH) from the pituitary gland, promoting the endocrine events leading to ovulation (Ortmann et al. 2002). In mares, 82–100% have been reported to ovulate within 48 h after histrelin treatment (Morrissey et al. 2019), and a dose of 250 µg IM is commonly used in commercial breeding programs (Cazales et al. 2023). More recently, administration of a reduced dose of 75 µg histrelin at the *Bai-hui* or Governing Vessel-1 acupoints was associated with high proportions of ovulation within 48 h, suggesting that lower doses may warrant further investigation (Cardoso et al. 2025).

Pharmacopuncture is the administration of a drug at an acupuncture point, aiming to combine the pharmacological action of the drug with the physiological effects of acupoint stimulation. This approach has been proposed as a strategy for reducing drug doses while maintaining therapeutic effects, with potential benefits including reduced adverse effects and treatment costs (Kim and Kang 2010).

In equine reproduction, reduced doses of hormonal agents administered at acupuncture points have previously been investigated. Alvarenga et al. (1998) reported that 10% of the conventional dose of prostaglandin F2α administered at the lumbosacral *Bai-hui* acupoint effectively induced luteolysis in mares. For ovulation induction, Ribeiro et al. (2020) administered 450 IU of human chorionic gonadotropin, approximately one-third of the conventional pharmacological dose, at Governing Vessel (GV)-1, resulting in 86.7% of mares ovulating within 48 h after treatment. *Bai-hui* has therefore been previously investigated for hormonal pharmacopuncture in mares, whereas other acupoints, including *Yan-chi*, have traditionally been indicated for reproductive disorders (Xie and Yamagiwa 2007), although their use for hormonal pharmacopuncture remains poorly investigated.

Although preliminary findings suggest that reduced histrelin doses may induce ovulation in mares, the efficacy of lower doses and the potential contribution of acupoint administration remain insufficiently investigated. In particular, whether a further reduction in histrelin dose can maintain an adequate ovulatory response and whether administration at reproductive acupoints provides an advantage over conventional intramuscular administration of the same reduced dose remain unclear. We hypothesized that reduced doses of histrelin would induce ovulation within 48 h in mares and that administration at the *Bai-hui* or *Yan-chi* acupoints would represent an effective approach for administering these reduced doses. Therefore, this pilot study aimed to evaluate reduced doses of histrelin (75 and 25 µg) for ovulation induction in mares and to compare conventional intramuscular administration with pharmacopuncture at the *Bai-hui* and *Yan-chi* acupoints.

## Materials and methods

### Study design and animals

This pilot study followed a partially randomized crossover design and was approved by the Animal Ethics Committee of the Pontifícia Universidade Católica do Paraná (PUCPR; protocol no. 3456051124). A convenience sample of mares from the teaching herd at the PUCPR Gralha Azul Experimental Farm was enrolled.

Eleven mares, aged 11 to 17 years, with body condition scores ranging from 4 to 6 according to the Henneke scoring system (Henneke et al. 1983), a history of normal reproductive function, and no evidence of reproductive or systemic disease were evaluated before enrollment. Under routine management, mares were kept in paddocks in groups of two to three on native pasture and received daily supplementation with 12 kg of Tifton hay, 1 kg of commercial concentrate feed (Pró Equino Tradição Pelleted, Agrária, Guarapuava, Paraná, Brazil), and 0.2 kg of mineral salt (Supra Sal Equine 80, Supra, Esteio, Rio Grande do Sul, Brazil), with water available *ad libitum*. The study was conducted during the Southern Hemisphere breeding season, from September 21, 2024 (spring equinox), to April 24, 2025. All mares underwent routine reproductive monitoring throughout the breeding season. Before enrollment, all mares had exhibited normal cyclic ovarian activity, including at least one confirmed ovulation during the season. Transrectal ultrasonographic examinations were performed regularly throughout the estrous cycle to monitor follicular development and determine the appropriate time for ovulation induction.

### Experimental protocols and treatments

Eight protocols were evaluated, with each mare scheduled to receive all eight protocols, one per estrous cycle (Supplementary Table 1): 250 µg histrelin IM (100% of the standard dose); 75 µg histrelin IM (30%); 25 µg histrelin IM (10%); no intervention; 1 mL saline at *Bai-hui*; 1 mL saline at *Yan-chi*; 25 µg histrelin at *Bai-hui*; and 25 µg histrelin at *Yan-chi*. Reduced histrelin doses were diluted in saline to a final volume of 1 mL. The no-intervention protocol served as the comparator for the other protocols.

The 250 µg IM protocol (250 µg in 1 mL) was administered to all mares during the first cycle and was not randomized. The sequence of the remaining seven protocols was determined by simple randomization using a manual draw conducted by an independent third party (Supplementary Table 2). Treatments were administered by investigators who did not perform the ultrasonographic examinations, and the examiners were unaware of the treatment administered. Mares were treated when they had a dominant follicle measuring 35–38 mm in two axes, a uterine edema score of 3, and a cervical relaxation score of 3 (Dascanio and McCue 2021), as assessed by transrectal palpation and ultrasonographic examination.

All intramuscular injections were administered at the base of the neck, corresponding to the region of the serratus ventralis cervicis muscle and superficial cervical fascia, using a 21 G 1/2 hypodermic needle. Administration at the acupuncture points was performed intramuscularly by a veterinarian certified in veterinary acupuncture, using an 18 G 1 ½-inch hypodermic needle at the *Bai-hui* acupoint and a 16 G 1/2-inch catheter at the *Yan-chi* acupoint.

The *Bai-hui* acupoint is located on the dorsal midline at the lumbosacral space. The *Yan-chi* acupoint is located midway between the tip of the coxal tuberosity and the *Sheng-peng* acupoint, which lies at the level of the fifth lumbar intervertebral space, lateral to the vertebral spinous process, in the region of the middle gluteal and longissimus dorsi muscles (Xie and Yamagiwa 2007). *Yan-chi* was always used ipsilateral to the dominant follicle. The anatomical locations of both acupoints are illustrated in Supplementary Fig. 1. *Bai-hui* was selected because it has previously been used for hormonal pharmacopuncture in mares (Alvarenga et al. 1998; Nie et al. 2001), whereas *Yan-chi* was selected based on its classical indication for reproductive disorders (Xie and Yamagiwa 2007).

### Reproductive monitoring and ovulation assessment

Treatments were administered at 18:00 h (hour 0). The first post-treatment ultrasonographic examination was performed 24 h later (18:00 h), followed by examinations twice daily at 10:00 h and 18:00 h (alternating intervals of 16 h and 8 h; i.e., at 24, 40, 48, 64, and 72 h, and so forth) until ovulation was detected. Ovulation was identified by the disappearance of the preovulatory follicle between two successive examinations and was therefore known only to have occurred within the interval between these examinations. The primary outcome was ovulation within 48 h, defined as ovulation detected at or before the 48-h examination; thus, no estimation of the exact time of ovulation was required. Ovulations first detected at the 24-h examination were excluded from the primary analysis on the assumption that they were more likely to represent spontaneous ovulations already in progress at the time of treatment. However, this assumption cannot be verified because spontaneous and rapidly induced ovulations cannot be distinguished with the present monitoring schedule. In the study by Morrissey et al. (2019), histrelin-induced ovulation occurred most frequently between 37 and 44 h after treatment, although 18.3% of mares ovulated at or before 36 h. Time to ovulation was neither estimated nor statistically compared; instead, ovulations were described according to the scheduled examination at which they were first detected. When ovulation was not detected within 48 h, examinations continued at the same intervals until detection.

### Statistical analysis

Statistical analyses included only the nine mares that completed all eight protocols; data from the two mares that did not complete the crossover study were excluded from all analyses. Pairwise comparisons of the proportion of mares ovulating within 48 h between each randomized treatment protocol and the negative control (no-intervention protocol) were performed using Fisher’s exact test. The non-randomized 250 µg IM protocol was considered a standard-dose reference and was not included in these inferential comparisons. Given the exploratory nature of this pilot study, p values were not adjusted for multiple comparisons and were interpreted accordingly. Exact 95% confidence intervals (CIs) for the proportion of mares ovulating within 48 h in each protocol were calculated using the Clopper–Pearson method. Normality of follicular diameter data was assessed using the Shapiro–Wilk test. Mean follicular diameters at treatment were compared among protocols using one-way repeated-measures analysis of variance (ANOVA), followed by Tukey’s multiple-comparison test. Statistical significance was set at *P* < 0.05. Statistical analyses were performed using GraphPad Prism, version 10.4.2 (GraphPad Software, San Diego, CA, USA).

## Results and discussion

Follicle diameter at induction (*p* = 0.851) did not differ among protocols. Nine of the 11 mares completed all eight protocols (18% loss). Two mares developed non-reproductive clinical conditions during the study period and subsequently ceased to exhibit regular ovarian cyclicity. Consequently, they did not complete the crossover study and were excluded from all analyses. One mare was last treated on 2 February 2025, after completing three protocols, and the other on 5 March 2025, after completing five protocols. The ovulation rates in each protocol are demonstrated in Table 1.

**Table 1:** Ovulation within 48 h after treatment, by protocol

Protocols	250 µg histrelin IM	75 µg histrelin IM	25 µg histrelin IM	No intervention	1 mL Saline Bai-hui	25 µg histrelin Bai-hui	1 mL Saline Yan-chi	25 µg histrelin Yan-chi
Treatments given, n	9	9	9	9	9	9	9	9
Ovulations detected at 24 h and excluded from the primary analysis, n	0	1	1	1	3	0	1	1
Ovulation within 48 h, % (n/N)	88.9% (8/9)	87.5% (7/8)	87.5% (7/8)	37.5% (3/8)	50.0% (3/6)	100.0% (9/9)	12.5% (1/8)	87.5% (7/8)
Exact 95% CI (%)	51.75–99.72	47.35–99.68	47.35–99.68	8.52–75.51	11.81–88.19	66.37–100	0.32–52.65	47.35–99.68
P value vs. no intervention	-----	0.119	0.119	-----	> 0.999	0.009	0.569	0.569

*n* number of observations, *CI* confidence interval, *IM* intramuscular. Statistical analyses included only the nine mares that completed all eight protocols. Ovulations first detected at the 24-h examination were excluded from the primary analysis, resulting in protocol-specific denominators. Exact 95% CIs were calculated using the Clopper–Pearson method. Ultrasonographic examinations were performed at 24, 40, 48, 64, and 72 h and twice daily thereafter until ovulation was detected. The 250 µg IM protocol was administered during the first cycle in all mares and was not randomized. Exploratory pairwise comparisons of each randomized protocol with the no-intervention protocol were performed using Fisher’s exact test. The 250 µg IM protocol was considered a non-randomized standard-dose reference and was not included in these inferential comparisons. P values were not adjusted for multiple comparisons.

**Fig. 1:**
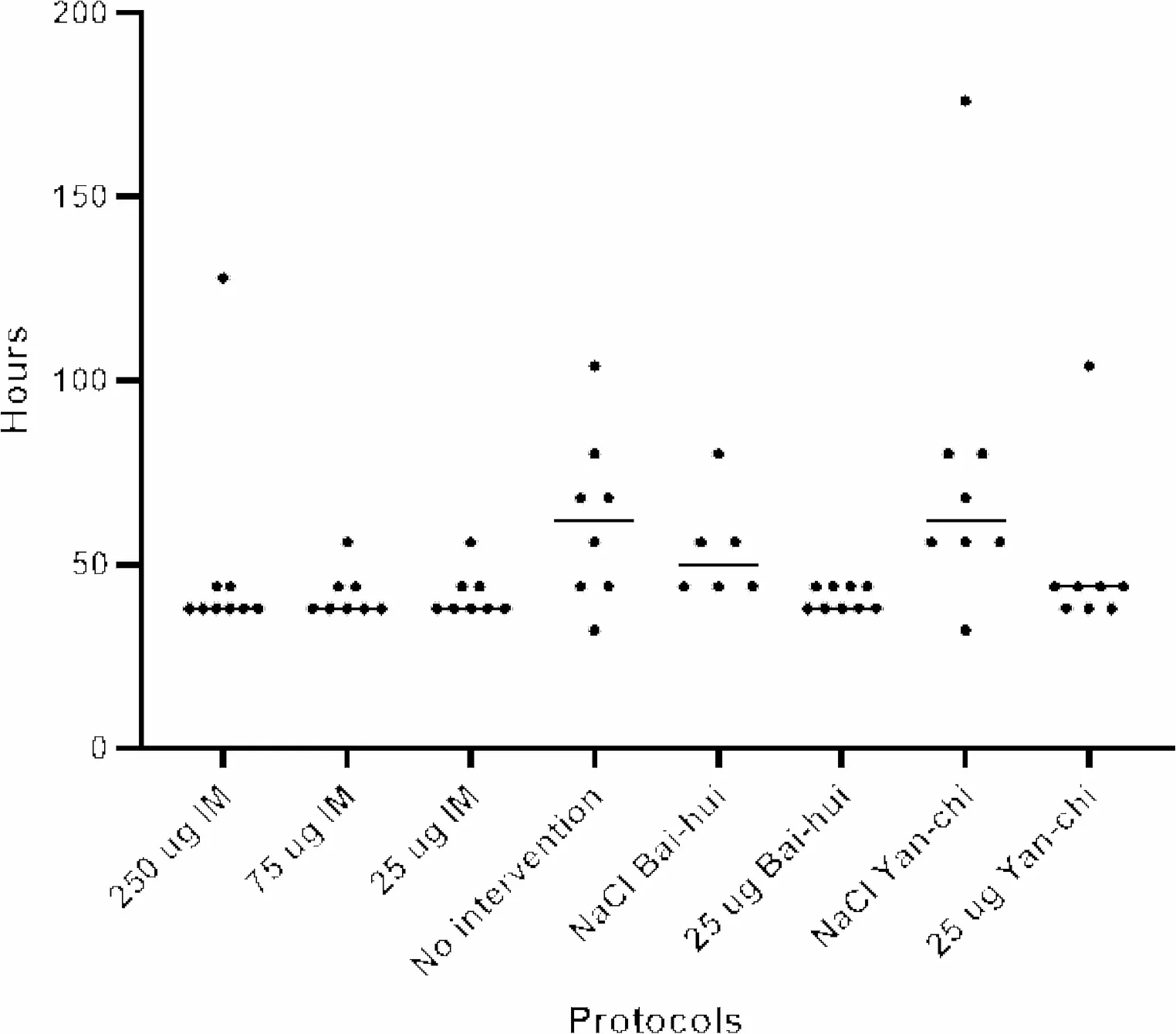
Examination at which ovulation was detected in each treatment, by protocol

Ovulation within 48 h occurred in 8/9 mares (88.9%) following 250 µg histrelin IM, 7/8 (87.5%) following 75 µg IM, 7/8 (87.5%) following 25 µg IM, 9/9 (100%) following 25 µg at *Bai-hui*, and 7/8 (87.5%) following 25 µg at *Yan-chi*. In comparison, ovulation within 48 h occurred in 3/8 mares (37.5%) with no intervention, 3/6 (50.0%) with saline at *Bai-hui*, and 1/8 (12.5%) with saline at *Yan-chi* (Table 1). Exact 95% CIs were wide, reflecting the small number of observations per protocol, and showed substantial overlap among protocols (Table 1). In the exploratory pairwise comparisons with the no-intervention protocol, 25 µg histrelin at *Bai-hui* was associated with a higher proportion of ovulations within 48 h (*p* = 0.009), whereas none of the remaining randomized protocols differed significantly from no intervention (*p* > 0.05). P values were not adjusted for multiple comparisons and should therefore be interpreted cautiously (Fig. 1).

In this exploratory study, ovulation within 48 h followed 87.5–100% of treatments with histrelin, including one-tenth of the standard dose, and 12.5–50.0% of treatments without drug, in line with the 82–100% reported for standard doses (Morrissey et al. 2019). This response is expected from the pharmacology of the drug (pituitary release of LH and FSH; Ortmann et al. 2002), and high proportions of ovulation within 48 h were observed when 25 µg was administered IM (7/8), at *Yan-chi* (7/8), or at *Bai-hui* (9/9). The numerically higher proportion at *Bai-hui* should not be interpreted as evidence of superiority: these protocols were not directly compared statistically, the confidence interval for *Bai-hui* (66.37–100%) overlaps those of the other histrelin protocols, and the small number of observations limits the precision of these estimates. *Bai-hui* was included because it has previously been investigated for hormonal pharmacopuncture in mares (Alvarenga et al. 1998; Nie et al. 2001), providing an experimental basis for its selection. In contrast, *Yan-chi* was selected primarily on the basis of its classical indication for reproductive disorders (Xie and Yamagiwa 2007), and evidence supporting hormonal pharmacopuncture at this point remains limited. The present data therefore do not demonstrate that administration at either acupoint adds to the pharmacological effect of histrelin, as proposed for pharmacopuncture (Kim and Kang 2010); this remains a hypothesis. This interpretation is consistent with the lack of advantage of *Bai-hui* over standard IM sites reported for prostaglandin (Nie et al. 2001) and with the similar ovulation rates reported following 75 µg histrelin at *Bai-hui* or GV-1 and 250 µg IM (Cardoso et al. 2025). Injection site, depth, and needle characteristics also differed among protocols and could have affected drug absorption independently of any acupuncture-specific effect. Further studies specifically designed to compare administration sites are required to determine whether acupoint selection contributes to the response beyond the pharmacological effect of histrelin itself. Ovulation rates without histrelin were 37.5%, 50.0%, and 12.5%, illustrating the variability associated with only 6–9 observations per protocol.

Several precautions were taken to minimize the risk of bias. Only mares with a healthy reproductive tract and regular estrous cycles were included, and strict criteria for ovulation induction were applied to promote comparability among protocols. The 8- and 16-h intervals between examinations did not allow the exact time of ovulation to be determined; therefore, differences in time to ovulation among protocols could not be assessed. Exclusion of ovulations detected at 24 h from the primary analysis was based on the assumption that these ovulations were more likely to have occurred spontaneously, although this cannot be verified with the present monitoring schedule. A sensitivity analysis including ovulations detected at 24 h as ovulations within 48 h did not materially change the interpretation of the results. The crossover design allowed each mare to receive all protocols, thereby reducing between-mare variability; however, potential carry-over effects cannot be excluded. Randomization of the sequence of seven of the eight protocols reduced the likelihood of a systematic association between these protocols and seasonal progression. However, the 250 µg IM protocol was administered first to all mares and was therefore confounded with treatment order and study period. Examiners performing the ultrasonographic assessments were blinded to treatment allocation, whereas the investigators administering the treatments could not be blinded.

The 250 µg IM protocol was always administered during the first cycle, beginning around the spring equinox, when some mares may still have been undergoing seasonal transition, and represented the first exposure to histrelin within the study. Its results are therefore potentially confounded by treatment order and seasonal progression and should be interpreted primarily as a reference rather than as a fully randomized comparison. Other limitations include the small sample size from a single herd, evaluation of only one estrous cycle per protocol, and the resulting limited statistical power and precision. Although the crossover design reduced interindividual variability and randomization of the remaining seven protocols reduced systematic differences related to treatment sequence, potential carry-over effects, seasonal variation, and physiological fluctuations across estrous cycles cannot be entirely excluded.

Two of the 11 mares did not complete the study after ceasing to exhibit regular ovarian cyclicity. Review of their clinical records showed that both mares developed concurrent non-reproductive clinical conditions during the study period. The cause of the altered cyclicity was not further investigated, and a causal relationship with either the concurrent clinical conditions or repeated histrelin exposure cannot be established. Although prolonged exposure to GnRH agonists has been associated with reduced pituitary responsiveness and delayed return to estrus following sustained-release deslorelin administration (McCue et al. 2002), the exposure pattern in the present study was substantially different. Nevertheless, a contribution of repeated GnRH-agonist administration cannot be excluded.

From a clinical perspective, the present findings provide preliminary evidence that reduced doses of histrelin, including one-tenth of the standard dose, may be sufficient to induce ovulation within 48 h in mares. However, the present study does not demonstrate an advantage of pharmacopuncture over conventional IM administration of the same reduced dose and therefore does not support recommending a specific route of administration. These findings warrant confirmation in a larger, fully randomized study with an appropriately calculated sample size, more frequent ultrasonographic monitoring, hormonal assessment, and follow-up of ovarian cyclicity. Such studies should also determine whether administration at specific acupoints provides any additional effect beyond that of the reduced histrelin dose itself. If pharmacopuncture is further investigated, potential effects of practitioner experience, injection technique, needle characteristics, and inter-operator variability should also be considered to establish the reproducibility and clinical applicability of these protocols.

## Supplementary Information

Below is the link to the electronic supplementary material.

Supplementary Material 1 (JPEG 116 KB)

Supplementary Material 2 (DOCX 14.6 KB)

Supplementary Material 3 (DOCX 17.9 KB)

## Data Availability

No datasets were generated or analysed during the current study.
