# Supplementary material for: Low-dose histrelin for ovulation induction in mares: a partially randomized crossover pilot study
Source: Vet Res Commun. 2026 Oct 20;51(1):8. doi: 10.1007/s11259-026-11583-9 (PMC13680520; doi:10.1007/s11259-026-11583-9)
Supplement: Supplementary file 1 [file 11259_2026_11583_MOESM2_ESM.docx]

Supplementary Table 1. Description of the ovulation induction protocols.
Protocol | Description | Dose and route/site

| **Protocol** | **Description** | **Dose and route/site** |
| --- | --- | --- |
| 1 | Histrelin (100% dose) | 250 µg - IM |
| 2 | Histrelin (30% dose) | 75 µg - IM |
| 3 | Histrelin (10% dose) | 25 µg - IM |
| 4 | No intervention | No intervention |
| 5 | Saline | 1 mL at *Bai-Hui* |
| 6 | Histrelin (10% dose) | 25 µg at *Bai-Hui* |
| 7 | Saline | 1 mL at *Yan-Chi* |
| 8 | Histrelin (10% dose) | 25 µg at *Yan-Chi* |

IM, intramuscular; µg, micrograms; mL, milliliters.
