# Supplementary material for: Low-dose histrelin for ovulation induction in mares: a partially randomized crossover pilot study
Source: Vet Res Commun. 2026 Oct 20;51(1):8. doi: 10.1007/s11259-026-11583-9 (PMC13680520; doi:10.1007/s11259-026-11583-9)
Supplement: Supplementary file 2 [file 11259_2026_11583_MOESM3_ESM.docx]

Supplementary Table 2. Sequence of treatment protocols assigned to each mare across estrous cycles. Protocol 1 was administered to all mares during the first cycle and was not randomized; the sequence of protocols 2–8 was randomized.

|  | **Cycle 1** | **Cycle 2** | **Cycle 3** | **Cycle 4** | **Cycle 5** | **Cycle 6** | **Cycle 7** | **Cycle 8** |
| --- | --- | --- | --- | --- | --- | --- | --- | --- |
| **Animal 1** | **1** | **2** | **6** | **8** | **5** | **3** | **4** | **7** |
| **Animal 2** | **1** | **7** | **4** | **3** | **8** | **6** | **5** | **2** |
| **Animal 3** | **1** | **5** | **3** | **4** | **2** | **7** | **8** | **6** |
| **Animal 4** | **1** | **3** | **8** | **7** | **6** | **4** | **2** | **5** |
| **Animal 5** | **1** | **6** | **5** | **2** | **7** | **8** | **3** | **4** |
| **Animal 6** | **1** | **4** | **7** | **5** | **3** | **2** | **6** | **8** |
| **Animal 7** | **1** | **8** | **2** | **6** | **4** | **5** | **7** | **3** |
| **Animal 8** | **1** | **5** | **4** | **7** | **6** | **8** | **2** | **3** |
| **Animal 9** | **1** | **4** | **3** | **2** | **5** | **6** | **7** | **8** |
| **Animal 10** | **1** | **2** | **7** | **6** | **3** | **4** | **8** | **5** |
| **Animal 11** | **1** | **3** | **8** | **5** | **7** | **2** | **6** | **4** |

1, 250 μg histrelin (intramuscular); 2, 75 μg histrelin (intramuscular); 3, 25 μg histrelin (intramuscular); 4, no intervention (control); 5, 1 mL saline at the Bai-hui acupoint; 6, 25 μg histrelin at the Bai-hui acupoint; 7, 1 mL saline at the Yan-chi acupoint; and 8, 25 μg histrelin at the Yan-chi acupoint.
